# Complete androgen insensitivity syndrome with cryptorchid sertoli cell tumor: a case report and literature review

**DOI:** 10.3389/fmed.2026.1936141

**Published:** 2026-09-15

**Authors:** Yuqing Wang, Jianfeng Huang, Wenxuan Yang, Jun Lei, Suping Li, Daiyu Zhu

**Affiliations:** Hubei Provincial Key Laboratory of Occurrence and Intervention of Rheumatic Diseases, Hubei Provincial Clinical Medical Research Center for Nephropathy, Minda Hospital of Hubei Minzu University, Hubei Minzu University, Enshi, Hubei, China

**Keywords:** androgen receptor, case report, complete androgen insensitivity syndrome, diagnosis, support cell tumor

## Abstract

**Objective:** Complete androgen insensitivity syndrome (CAIS) is a 46,XY disorder of sex development caused by mutations in the androgen receptor (AR) gene. This article reports a case of a patient clinically diagnosed with CAIS complicated by a cryptorchid primary borderline Sertoli cell tumor, who presented to Minda Hospital of Hubei Minzu University in 2024. The patient exhibited a female phenotype and presented with primary amenorrhea. The diagnosis was established based on clinical manifestations, endocrine evaluation, imaging studies, and chromosomal karyotype analysis. The patient underwent laparoscopic bilateral gonadectomy, followed by postoperative estrogen replacement therapy. Postoperative histopathological examination revealed a cryptorchid borderline Sertoli cell tumor. The clinical presentation of CAIS is insidious, which may lead to delayed diagnosis. The identification of a Sertoli cell tumor in this case highlights that the risk of gonadal neoplasia should not be overlooked. Strengthening early recognition, appropriately timing gonadectomy, and ensuring long-term follow-up are of considerable importance for patient management.

## 1 Introduction

Complete androgen insensitivity syndrome (CAIS) represents the most severe phenotype within the spectrum of androgen insensitivity syndrome (AIS). It is a rare X-linked recessive disorder characterized by 46,XY disorders of sex development (DSD), with an estimated occurrence rate of approximately 1 in 20,000 to 1 in 64,000 live births (1). Complete Androgen Insensitivity Syndrome (CAIS) is a form of 46,XY disorder of sex development (DSD) caused by functional abnormalities of the androgen receptor (AR). Affected individuals possess a 46,XY karyotype and testicular tissue, yet present with a female phenotype. The diagnosis is primarily based on clinical and biochemical features, including a 46,XY karyotype, female external genitalia, absence of Müllerian duct structures, presence of testes, and normal or elevated testosterone and luteinizing hormone (LH) levels. Detection of pathogenic variants in the AR gene can provide further molecular confirmation (2). The risk of testicular tumor development in patients with CAIS progressively increases after puberty, rendering the optimal timing of gonadectomy a persistent point of clinical attention and debate. Although the majority of resected gonadal specimens demonstrate benign pathological findings, the occurrence of rare lesions, such as the synthesis of sex cord-stromal tumors, is infrequently reported. Herein, we present a case of a cryptorchid borderline Sertoli cell tumor diagnosed postoperatively via pathological examination. By analyzing the clinical characteristics, diagnostic and therapeutic course, and follow-up outcomes of this case, in conjunction with a review of the relevant literature, this report aims to discuss the risk of neoplasia, strategies for gonadal management, and the importance of long-term surveillance in patients with CAIS.

## 2 Case presentation

A 19-year-old phenotypic female patient was referred for evaluation of primary amenorrhea. Denying the history of sexual life in the past. Physical examination revealed a female body habitus with no thyroid cartilage prominence, height 167 cm, weight 70 kg, Tanner stage 2 breast development (Grade I), poor nipple development, sparse pubic hair, normal labia majora, underdeveloped labia minora with congested medial surfaces, and a vaginal depth of approximately 7 cm. No masses were palpable in either inguinal region. Digital rectal examination revealed no palpable masses within the pelvic cavity. Auxiliary examinations included pelvic color Doppler ultrasound, which showed a cystic mass in the left pelvis (suggestive of testicular cystic degeneration; Figure 1), a hypoechoic area in the right inguinal region (suggestive of cryptorchidism), and no evident uterine echo within the pelvic cavity (Figure 2). Urinary system ultrasound was unremarkable. Pituitary and sellar region MRI showed no abnormalities. The patient's karyotype was 46,XY. Following admission, laparoscopic bilateral cryptorchidectomy was performed. Laparoscopic examination revealed no uterus, fallopian tubes, or ovaries; a cystic tumor was observed in the left pelvis, and a gray-white tumor was identified to the right of the iliac bone (Figure 3). Intraoperative frozen section pathological examination suggested cryptorchidism and a sex cord-stromal tumor lesion, with malignancy not excluded. Consequently, omentectomy and multi-point peritoneal biopsy were performed. Final pathological examination of the surgical specimen revealed the following: (Left gonad) 1. Microscopically, the tissue was identified as testicular tissue, with immature spermatogenic cells within the seminiferous tubules and no evidence of spermatogenesis, consistent with a diagnosis of cryptorchidism. Immunohistochemical findings: 4# Ki67 (low proliferative index), OCT3/4 (−), SALL4 (−). 2. Sertoli cell tumor, ICD-O code: 8640/1 (note: 8640/1 denotes a borderline tumor). Immunohistochemical findings: CK20 (−), CEA (−), CK7 (−), CR (+), E-cadherin (−), Inhibin-α (+), Ki67 (low proliferative index), Napsin A (−), P16 (scattered +), P53 (wild-type), PAX-8 (−), PCK (+), PR (+), SALL4 (−), vimentin (+), WT-1 (+). The tumor measured 5.0 cm in greatest dimension and exhibited a cystic-solid cut surface, with mucinous contents within the cystic component and gray-yellow, firm solid areas. Microscopically, the tumor cells displayed complex proliferation with mild-to-moderate cytologic atypia, rare mitotic figures, no definitive stromal invasion, and no evidence of tumor cell infiltration into the surrounding tissues (Figure 4), and the right gonad was testicular tissue. No metastatic lesions were found in the omentum or peritoneum. Postoperative assessment indicated no need for additional therapy, and estradiol valerate was prescribed for estrogen supplementation. Regular follow-up showed that serum estradiol levels fluctuated between 52.46 and 76.86 pg/mL, with no abnormalities detected on imaging studies.

**Figure 1:**
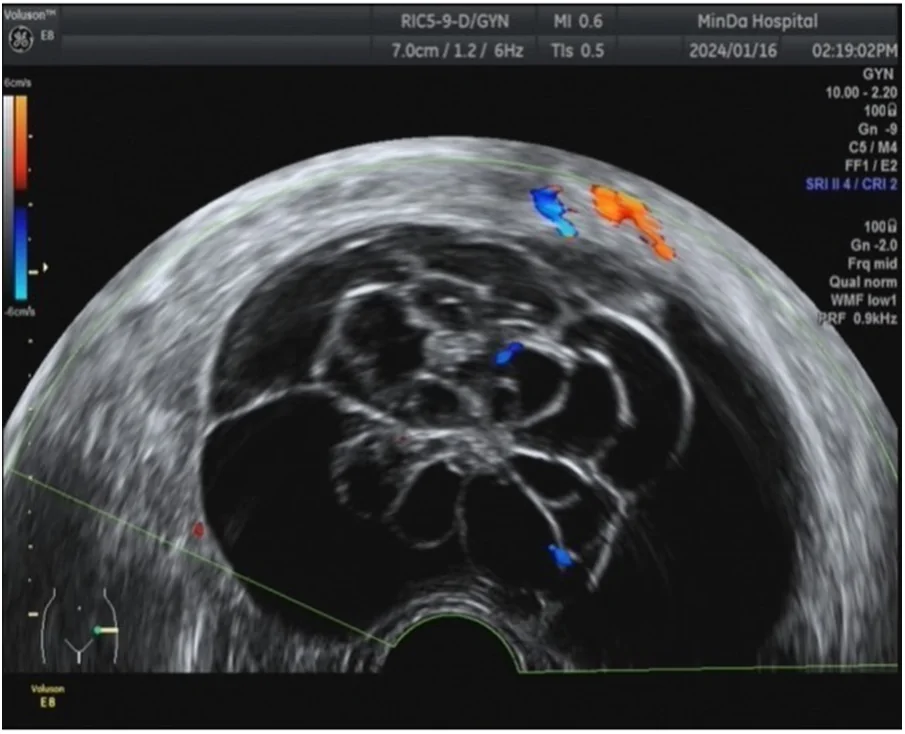
Pelvic ultrasound findings of the enlarged left undescended testis.

**Figure 2:**
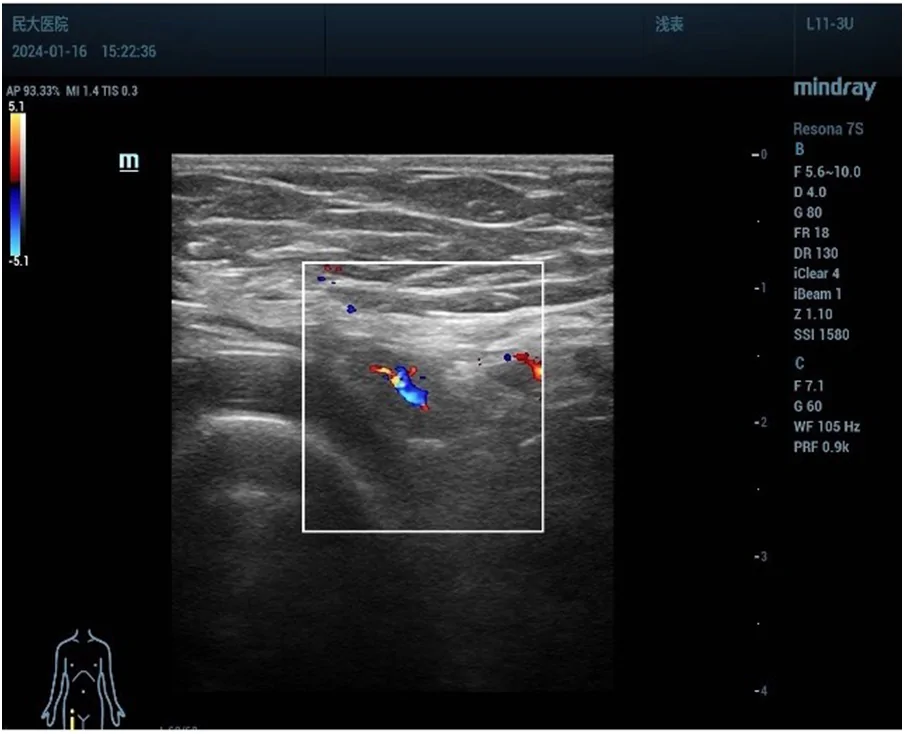
Ultrasound findings of the right inguinal undescended testis.

**Figure 3:**
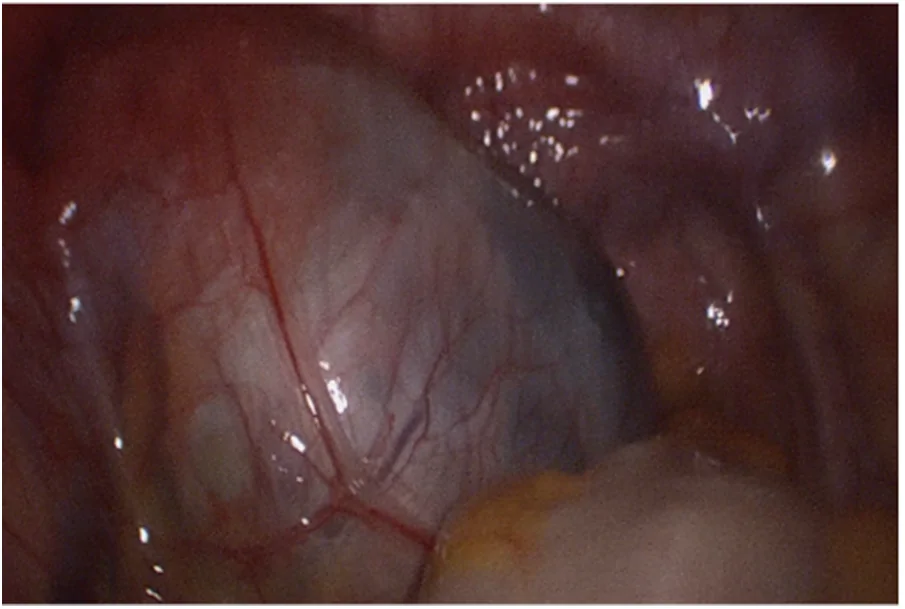
Laparoscopic findings of the enlarged left undescended testis.

**Figure 4:**
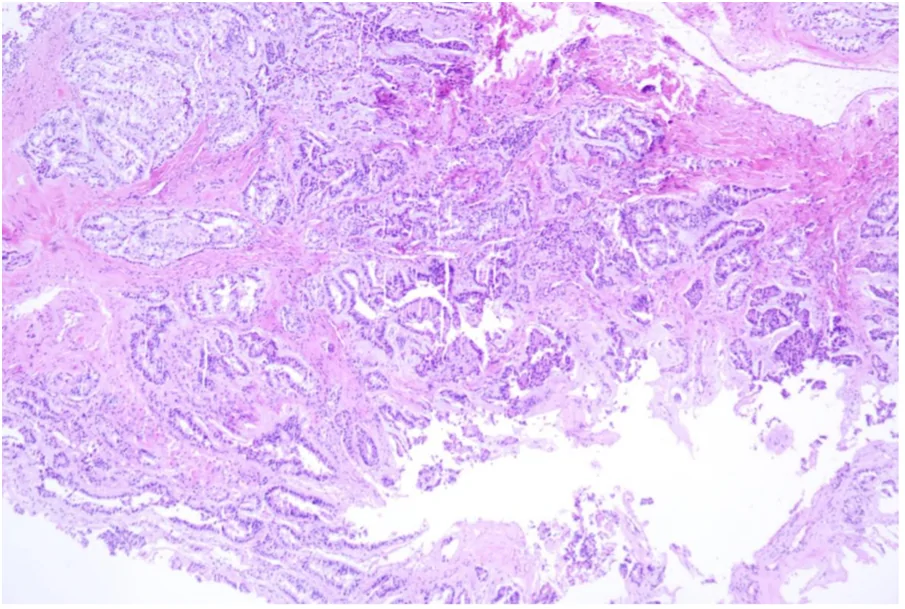
Histopathological and immunohistochemical findings of the left gonad.

## 3 Discussion

In this study, the patient presented with primary amenorrhea and clinical features including congenital absence of the uterus, female phenotype, 46,XY karyotype, inguinal cryptorchidism, and extremely elevated AMH levels (3, 4).

Androgen insensitivity syndrome (AIS) is an important subtype of 46,XY disorders of sex development (46,XY DSD). 46,XY DSD comprises a heterogeneous group of disorders, and partial androgen insensitivity represents one of the most important causes of 46,XY DSD. AIS is further classified into three subtypes: complete androgen insensitivity syndrome (CAIS), partial androgen insensitivity syndrome (PAIS), and mild androgen insensitivity syndrome (IAIS). This disorder follows an X-linked recessive inheritance pattern and is caused by mutations in the androgen receptor (AR) gene, which is located at Xq11-12 on the proximal long arm of the X chromosome, near the centromere. The mothers of affected individuals are typically carriers of the pathogenic variant, whereas the affected individuals are usually individuals with a 46,XY karyotype (5).Because the patient wished to protect personal privacy and declined our offer of free ARgene sequencing, the specific pathogenic gene variant could not be determined. Among patients with a confirmed diagnosis of CAIS, ARgene mutations account for the majority of cases. To date, over 1,000 types of ARgene mutations have been identified. The most common types of molecular variants include missense variants, nonsense variants, insertion-deletion variants, splice site variants, and trinucleotide repeat expansion variants (6–8). Studies have indicated that different phenotypes may occur within the same family lineage. For patients with suspected familial aggregation tendencies, genetic testing and genetic counseling should be pursued whenever possible (9).

The hormonal phenotype observed in this patient may represent an endocrine manifestation of CAIS complicated by a Sertoli cell tumor rather than an atypical feature of CAIS alone (10). Longitudinal pre-gonadectomy assessments revealed a progressive hormonal shift from adolescence to early adulthood, characterized by a decline in testosterone from 70 ng/dL at approximately 15 years of age to 49.09 ng/dL at 19 years, accompanied by an increase in estradiol from 51 to 183.66 pg/mL and a decrease in LH from 1.72 to 0.20 IU/L. Enhanced aromatase activity has been described in Sertoli cell tumors and may facilitate the conversion of androgens to estrogens, resulting in increased estradiol levels and subsequent suppression of gonadotropin secretion through negative feedback (10, 11). Notably, a comparable pattern of low testosterone, elevated estradiol, and suppressed gonadotropins has been reported in CAIS associated with a Sertoli cell tumor (10). Thus, the progressive hormonal shift in our patient may reflect the gradual development and increasing endocrine activity of the borderline Sertoli cell tumor, although hormonal changes alone cannot establish tumor growth kinetics. These findings highlight that new or progressive hormonal alterations after puberty in patients with CAIS, particularly declining testosterone accompanied by rising estradiol and suppressed gonadotropins, should raise suspicion for gonadal neoplasia and warrant further evaluation (10, 12).

Why do patients with CAIS develop tumors? The underlying reasons are as follows: in patients with CAIS, the testes are retained in the abdominal cavity or inguinal region and are chronically exposed to a relatively high-temperature environment, which can lead to abnormalities in testicular tissue and germ cell maturation (13). Meanwhile, the complete loss of androgen receptor function disrupts androgen signaling, resulting in delayed germ cell maturation within the testes and the persistence of cells exhibiting a primordial germ cell-like phenotype. In addition, patients with CAIS are subject to long-term abnormalities in the sex hormone environment and testicular microenvironment. Recent molecular studies have suggested that alterations in testicular somatic cell states and changes in the testosterone/estradiol ratio may be involved in modulating the risk of germ cell tumors. Some of the persistently immature germ cells may further develop pre-GCNIS and GCNIS-like changes and may progress with age into invasive testicular germ cell tumors. Sustained hormonal stimulation of the gonadal stroma may also be associated with the development of Sertoli cell-derived tumors (12, 14–16). The final pathological examination in the present study revealed a cryptorchid borderline Sertoli cell tumor. Regarding histological characteristics, a study as early as 1992 proposed that the embryonic gonad arises from the proliferation of coelomic epithelial cells on the medial side of the mesonephros and the primordial mesenchyme, forming two elevated genital ridges. Testicular sex cord-stromal tumors originate from the primitive germinal epithelium and mesenchyme of the genital ridge. Further differentiation of these components yields Sertoli cells and Leydig cells in the testis, whereas in the ovary, they differentiate into granulosa cells and theca cells. The sex cord-stromal components of both sexes are derived from the primitive germinal epithelium and mesenchyme (17). At the microscopic level, these tumors can be classified into two types. In the common type, tumor cells are primarily arranged in solid or hollow tubules, or in cords, with a basilar membrane surrounding the tubules; the tumor cells are cuboidal, with pale or eosinophilic cytoplasm, mild nuclear morphology, minimal atypia, rare mitotic figures, and the stroma consists of fibrous connective tissue. The sclerosing type is characterized by extensive stromal hyalinization, with tumor cells scattered in a cord-like pattern. The large cell calcifying type features tumor cells with abundant, eosinophilic cytoplasm, accompanied by myxoid degeneration of the stroma and characteristic calcifications. Malignant cases may exhibit marked cellular atypia, increased mitotic activity, necrosis, and vascular invasion (18). Due to the rarity and uniqueness of CAIS patients with cryptorchid primary borderline Sertoli cell tumor, this article also summarizes some similar cases over the past 10 years (Table 1).

**Table 1:** Clinical and pathological characteristics, gonadal neoplasms, treatment, and outcomes of patients with complete androgen insensitivity syndrome reported in the literature.

Study/Case	Age; AIS subtype and molecular confirmation	Gonadal location	Pathological diagnosis	Malignant features	Treatment and outcome
Karseladze et al., 2024	15 years; CAIS. 46,XY; NGS identified a pathogenic AR c.77delT, p.(Leu26Argfs*8) frameshift deletion and a likely pathogenic RAC1 p.A94 V variant.	Bilateral pelvic gonads, near the pelvic brim	Bilaterally, Sertoli cell hamartomas (SCHs) and Sertoli cell adenomas (SCAs), with a left-sided Sertoli–Leydig cell tumor (SLCT); left paratesticular leiomyoma and bilateral rudimentary fallopian tubes were also identified.	No definite malignant features. Ki-67 ≤ 4%; germ-cell tumor markers were negative, and no *in situ* or invasive germ-cell neoplasia was identified. No definite malignant histological features were reported for the SLCT.	Laparoscopic bilateral gonadectomy. Case-specific follow-up duration was not reported.
Yildiz et al., 2026	60 years; CAIS. Previously diagnosed during adolescence for primary amenorrhea; 46,XY; AR molecular testing was not reported.	Bilateral pelvic/adnexal regions; approximately 4.5 × 3.5 cm on the left and 3 cm on the right	Left gonad: Sertoli cell tumor, Leydig cell tumor, and sclerotic seminiferous tubules; right gonad: Sertoli cell adenoma (SCA).	Predominantly benign/low-grade proliferative lesions; no necrosis, marked atypia, or metastasis was reported.	Laparoscopic bilateral gonadectomy. No postoperative recurrence or metastasis was reported; long-term surveillance was recommended.
Yu & Liu, 2024 (Case 2)	16 years; CAIS. 46,XY; diagnosis based on clinical, hormonal, and imaging findings; no AR molecular testing was reported.	Bilateral adnexal/pelvic gonads	Bilaterally, Leydig cell hyperplasia with seminiferous tubules containing predominantly Sertoli cells; no definite tumor identified.	No malignant tumor identified.	Laparoscopic bilateral gonadectomy followed by estrogen replacement therapy; no tumor recurrence was reported during follow-up.
Izawa et al., 2021 (10)	8 years 9 months at presentation; gonadectomy at 10 years 4 months; CAIS. 46,XY,inv(9)(p12q13); a hemizygous AR p.Pro893Leu missense variant confirmed the diagnosis.	Left inguinal gonad and right intra-abdominal gonad	Right gonad: Sertoli cell tumor; left gonad: immature seminiferous tubules predominantly composed of Sertoli cells, consistent with abnormal testicular development in CAIS.	No pathological evidence of malignancy. The tumor was encapsulated and lacked malignant histological features; no malignant changes were observed during the 19-month preoperative surveillance period.	Bilateral gonadectomy at 10 years 4 months because of progressive tumor enlargement and marked estradiol elevation. Estradiol rapidly decreased after surgery; no recurrence was reported.
Thirunavukkarasu et al., 2016	17 years; CAIS. 46,XY; diagnosis based on female phenotype, absent uterus, undescended gonads, and karyotype; no AR molecular testing was reported.	Bilateral pelvic gonads; approximately 5 cm on the right and 3 cm on the left	Right gonad: classical seminoma with adjacent intratubular germ cell neoplasia; left gonad: Sertoli cell adenoma.	Definite malignancy: right-sided seminoma. PLAP and CD117 were positive; histopathology confirmed classical seminoma.	Bilateral pelvic gonadectomy/mass excision followed by low-dose estrogen replacement. No chemotherapy was administered; no recurrence was observed during 9 months of follow-up.

CAIS, complete androgen insensitivity syndrome; AIS, androgen insensitivity syndrome; AR, androgen receptor; NGS, next-generation sequencing; SCH, sertoli cell hamartoma; SCA, sertoli cell adenoma; SLCT, sertoli–leydig cell tumor; ITGCN, intratubular germ cell neoplasia; NR, not reported. Yu & Liu (2024) Case 1 was 46,XY pure gonadal dysgenesis and was therefore excluded; only Case 2 was included. Information not reported in the source articles was marked as NR rather than inferred. *In HGVS nomenclature, the asterisk () denotes a termination codon (stop codon). In the variant p.(Leu26Argfs8), the “fs” indicates a frameshift mutation, while “8” indicates that the frameshift results in a premature termination codon eight amino acids downstream of the frameshift site.

Research indicates that the risk of developing such malignant tumors before puberty is reduced. In patients with complete androgen insensitivity syndrome (CAIS), the risk of gonadal gonadal malignant tumors in adult CAIS patients is estimated to be 14% (range 0%–22%), while the risk in childhood is only 0.8% to 2.0% (19). The aforementioned patients were subjected to gonadectomy following hospitalization. Currently, there are two opposing viewpoints internationally regarding the optimal timing of gonadectomy: one advocates for postpubertal resection, with the advantage that endogenous testosterone production by the testes can promote the development of secondary sexual characteristics (20). The other supports early resection, with the advantage of reducing the risk of tumorigenesis (21). In the present study, the patient was diagnosed with a cryptorchid borderline Sertoli cell tumor of the testis after puberty. For patients diagnosed with CAIS at an early stage who do not wish to undergo early gonadectomy, close monitoring throughout puberty, for example, inhibin B measurement reflects Sertoli cell number and spermatogenic status, and serum inhibin B can be regarded as an auxiliary biomarker for Sertoli cell and gonadal function in CAIS patients with retained gonads; however, its utility in detecting gonadal tumors has not been established. Döhnert et al. proposed that follow-up for CAIS patients with retained gonads may be conducted every 6 months, including imaging studies (inguinal/scrotal testicular ultrasound, pelvic and abdominal MRI), tumor markers (e.g., AFP, β-HCG, CA125, LDH), and hormonal assessment, with hormonal assessment including FSH, LH, testosterone, and inhibin B. However, in the studies by Deans, R. and Nordenström, A., it was proposed that the follow-up interval could be extended to one year, and it was further recommended that bone mineral density monitoring be added. Gonadectomy may be performed at an appropriate time based on dynamic observation of the aforementioned medical indicators; nevertheless, it is clear that no single examination currently available can reliably detect early GCNIS or precancerous lesions (19, 22, 23).

After surgery, the patients in this study received estrogen replacement therapy, which serves both to maintain female secondary sexual characteristics and to effectively prevent osteoporosis. However, the adverse effects of estrogen, including night sweats, insomnia, and hot flashes, may lead patients to discontinue medication on their own; therefore, long-term follow-up monitoring of hormone levels and bone mineral density is required. Prior to admission, the patients had not received psychological counseling. At discharge, they were advised to seek consultation in the psychology department to enhance their gender identity and thereby improve quality of life. During follow-up, the patients demonstrated good compliance, attending regular outpatient follow-up visits in our department as well as scheduled consultations in the psychology department of our hospital, where they received psychological support.

## 4 Conclusion

In summary, CAIS is a rare disorder of sex development. This study reports a case of CAIS complicated by cryptorchid Sertoli cell tumor. The identification of this neoplastic lesion further underscores the importance of monitoring cryptorchidism and individualized gonadal management.

## Data Availability

The original contributions presented in the study are included in the article/Supplementary Material, further inquiries can be directed to the corresponding author.
