# Correction: Complete androgen insensitivity syndrome with cryptorchid sertoli cell tumor: a case report and literature review

**DOI:** 10.3389/fmed.2026.2019594

**Published:** 2026-10-16

**Authors:** Yuqing Wang, Jianfeng Huang, Wenxuan Yang, Jun Lei, Suping Li, Daiyu Zhu

**Affiliations:** Hubei Provincial Key Laboratory of Occurrence and Intervention of Rheumatic Diseases, Hubei Provincial Clinical Medical Research Center for Nephropathy, Minda Hospital of Hubei Minzu University, Hubei Minzu University, Enshi, Hubei, China

**Keywords:** androgen receptor, case report, complete androgen insensitivity syndrome, diagnosis, support cell tumor

The funder Minda Hospital of Hubei Minzu University Open Research Project, No.OIR202406Y to Daiyu Zhu was erroneously omitted.

The original version of this article has been updated.

